# Epigenetic Alterations in Anesthesia-Induced Neurotoxicity in the Developing Brain

**DOI:** 10.3389/fphys.2018.01024

**Published:** 2018-07-31

**Authors:** Ziyi Wu, Ping Zhao

**Affiliations:** Department of Anesthesiology, Shengjing Hospital of China Medical University, Shenyang, China

**Keywords:** anesthetic agents, developmental neurotoxicity, DNA methylation, histone modification, non-coding RNAs

## Abstract

Before birth and early in life, the developing brain is particularly sensitive to environmental and pharmacological influences. Increasing experimental evidence suggests that an association exists between exposure to anesthesia during a vulnerable period of brain development and subsequent poor neurodevelopmental outcomes. However, the mechanisms underlying this association are not fully understood. Epigenetics, broadly defined as the regulation of gene expression without alterations of DNA sequence, has become a field of tremendous interest in neuroscience. In recent years, a growing body of literature suggests that anesthesia-induced long-term changes in gene transcription and functional deficits in learning and behavior later in life are mediated via epigenetic modifications. This brief review provides an overview of epigenetic mechanisms and highlights the emerging roles played by epigenetic dysfunctions in the processes of anesthesia-induced neurotoxicity in the developing brain. Epigenetic targeting of DNA methyltransferases and/or histone deacetylases may have some therapeutic value. Epigenetics may lead to the identification of novel markers that contribute toward considerable translational significance in the field of neuroprotection.

## Introduction

Every year, millions of pregnant women, neonates, infants, and toddlers across the world are exposed to anesthesia for surgeries, therapeutic procedures, and imaging studies. However, before birth and early in life, the developing brain is a particularly sensitive target to environmental and pharmacological influences. Currently the majority of general anesthetics used function through *N*-methyl-D-aspartate (NMDA) receptor and/or γ-aminobutyric acid receptor type A (GABA_A_) receptor modulation (Garcia et al., 2010; Petrenko et al., 2014). Inhaled and intravenous anesthetics share overlapping effects on these two receptors (Hudson and Hemmings, 2011). Prolonged or excessive stimulation of NMDA receptors and/or GABA_A_ receptors may interfere with the neural circuitry during early neurodevelopment, a consequence that may account for the developmental neurotoxicity induced by general anesthetics (Fredriksson et al., 2004; Fredriksson et al., 2007). Accumulating evidence from rodent and primate studies has demonstrated that in utero [most general anesthetics can cross the placenta and reach fetal blood (Palanisamy, 2012)] or neonatal exposure to commonly used inhaled and intravenous general anesthetics is associated with neural degeneration and subsequent neurocognitive impairments, manifested in learning and memory disabilities (Andropoulos and Greene, 2017). Several retrospective clinical studies have demonstrated that childhood exposure to general anesthetics pose an increased risk of neurocognitive impairments (Creeley, 2016). Anesthesia-induced toxic effects on the central nervous system attract wide public attention but their underlying mechanisms are largely unknown. An increasing number of studies demonstrate that general anesthetics may initiate abnormal neurodevelopment, at least in part, through epigenetic mechanisms.

Epigenetics refers to the study of heritable changes in the expression and function of genes without alterations in DNA sequence. Major epigenetic mechanisms include DNA methylation, histone modification, and non-coding RNAs. Epigenetics, which acts as a mediator between genotype and environment, plays significant roles in brain development and cognitive processes by translating environmental cues into changes in the expression of target genes (Van Soom et al., 2014; Kundakovic and Champagne, 2015). Emerging studies have revealed that epigenetic dysregulation is one of the hallmarks of abnormal brain function and neurodegenerative diseases (Delgado-Morales et al., 2017; Tran and Miyake, 2017). It is especially interesting that epigenetic dysregulation currently garners much attention as a pivotal player in anesthesia-induced neurotoxicity at the early stages of brain development.

## Epigenetic Changes and Therapeutic Approaches for Anesthetic-Induced Developmental Neurotoxicity

### DNA Methylation and DNMTs Inhibitors

DNA methylation (Moore et al., 2013; Heyward and Sweatt, 2015) is the most characterized epigenetic event in which DNA methyltransferases (DNMTs) catalyze the covalent conversion of cytosine residues to 5-methylcytosine residues, which can lead to long-term down-regulation of target genes. Three active mammalian DNMTs have been identified, DNMT1, DNMT3a, and DNMT3b. DNMT1 has a preference for hemi-methylated DNA, whereas DNMT3a and DNMT3b are involved in the formation of new methylation patterns to unmodified DNA, called *de novo* DNA methylation. DNA methylation regulates gene expression by recruiting proteins involved in gene repression and/or by blocking promoter regions to which activating transcription factors should bind. DNMT inhibitors are widely used as epigenetic modulators, thereby representing promising targets in epigenetic therapies. DNMT inhibitors can modulate aberrant DNA methylation pattern in a reversible manner by inhibiting DNMT activity.

A recent study has demonstrated that the expression of DNMT1 is significantly increased in the hippocampi of rats with neonatal exposure to isoflurane (Wu et al., 2016). A further chromatin immunoprecipitation (ChIP) study has revealed increased occupancy and methylation (5′-cytosine) levels at the promoter region of the neurotrophin, brain-derived neurotrophic factor (BDNF) gene, a critical modulator of synaptic plasticity. This increased methylation at the BDNF promoter region was associated with suppression of BDNF expression and subsequent memory loss. Hippocampal DNMT3a and DNMT3b levels are increased in a rat model with repeated neonatal sevoflurane exposure, resulting in the hypermethylation of BDNF and Reelin genes (Ju et al., 2016). Interestingly, DNMT1 levels do not significantly change (Ju et al., 2016). Different model species, anesthetics, and/or exposure doses within these studies may account for these findings. Pretreatment with the DNMT inhibitor, 5-aza-2′-deoxycytidine, reverses sevoflurane-induced dendritic spine decreases and cognitive abnormalities by inhibiting DNMT activity and enhancing the expression of synaptic plasticity-related genes (Ju et al., 2016).

### Histone Modifications and HDAC Inhibitors

There have been some important publications in recent years that have pointed out the importance of histone modifications in neural development and brain function (Keverne, 2014; Sen, 2015). Histone modifications encompass a vast variety of post-translational modifications to the tails of histone proteins, and these give rise to varying cellular outcomes. In particular, histone modification by acetylation, which involves the addition of an acetyl group to lysine residues present at the N-terminal tails of the nucleosome, is the most extensively studied one in neuroscience. Generally, acetylated histones are associated with increased transcriptional activity, whereas deacetylated histones are associated with decreased transcriptional activity. Histone acetylation is mediated through histone acetyltransferases (HATs) and histone deacetylases (HDACs), each family comprised of several isoforms. HDACs reverse the activity of HATs and cause a decrease in transcription through the removal of acetyl groups from histone tails. HDACs are typically grouped into four classes: class I HDACs (1–3 and 8), class II HDACs (4–7, 9, and 10), class III HDACs (also known as sirtuins, which are structurally NAD^+^ dependent for enzymatic activity) and class IV HDACs (referred to as HDAC11). Unfortunately, dysregulation of the HATs/HDACs balance may lead to pathologies which have been implicated in anesthesia-induced neurological disorders.

As histone acetylation is typically associated with an increase in the expression of numerous neural genes and in turn, plays an important role in synaptic plasticity, learning and memory, it is generally considered favorable for memory and cognition. As well as enhancing HAT activity, HDAC inhibitors, which are predominantly used as anticancer drugs, have recently been suggested to act as neuroprotective agents and are emerging as powerful cognitive enhancers. HDAC inhibitors are therefore a novel therapy to treat cognitive impairments that are linked to a wide range of neurodegenerative and psychiatric disorders (Graff and Tsai, 2013; Ganai et al., 2016).

In previous studies, rodents exposed to anesthetics during the gestational or neonatal period exhibited long-term developmental neurocognitive abnormalities and alterations in histone acetylation. For example, hippocampal levels of HDAC3 and HDAC8, but not HDAC1 and HDAC2, were elevated in adult rats that were exposed to sevoflurane in the neonatal period. Moreover, sevoflurane-exposed rats showed reduced hippocampal levels of acetylated H3K9/14 and H4K5/12 and reduced expression of several genes involved in neurodevelopment and neuroplasticity including BDNF, c-Fos, and postsynaptic density protein 95 (Psd-95). It is worth noting that sevoflurane exposure was associated with changes in specific brain regions, such as decreased H3K9 and H4K5/12 acetylation in the hippocampal CA1 region, as well as decreased H2K14 acetylation in both that hippocampal CA1 and dentate gyrus (DG) regions. Moreover, impaired hippocampus-dependent spatial and associated memory is observed, rather than explorative behaviors. Upregulation of histone acetylation pharmacologically, using the HDAC inhibitor sodium butyrate (NaB), ameliorated the developmental side effects caused by sevoflurane exposure (Jia et al., 2016). Increased HDAC2 activity and decreased acetylation of H3 but not H4 is observed in the hippocampus of isoflurane-exposed rats and in isoflurane-exposed hippocampal neurons, along with decreased histone acetylation of hippocampal neurons in the promoter regions of GLT-1 and mGLuR1/5. NaB improves cognitive impairments *in vivo* by restoring a decrease in histone acetylation of glutamatergic systems, which has been confirmed in hippocampal neurons (Liang and Fang, 2016). Trichostatin A (TSA) has also been shown to offer protection against neurocognitive impairment and abnormal hippocampal histone acetylation in isoflurane-exposed mice during the neonatal period by enhancing histone acetylation and downstream c-Fos gene expression (Zhong et al., 2015).

The cyclic-AMP-response element binding protein (CREB) signaling pathways have been implicated in anesthesia-induced neurodegenerative changes in basic experimental studies (Bi et al., 2016; Ding et al., 2017). CREB-binding protein, also known as CBP, functions by activating transcription, as a co-activator of the transcription factor CREB. CBP is also characterized as a HAT, regulating the degree of histone acetylation via its intrinsic HAT domain. A general anesthetic (a sedative dose of midazolam followed by a combination of nitrous oxide and isoflurane) causes fragmentation of CBP with decrease in its HAT activity. Hypoacetylated H3 results in down-regulated transcription and expression of BDNF and c-Fos (Dalla Massara et al., 2016). ChIP assays have revealed that the levels of acetylated H3 in CREB binding sites at the promoter regions of BDNF and c-Fos genes are decreased in the hippocampus, which in turn inhibits their transcription. Reversal of histone hypoacetylation with NaB blocks the morphological and functional impairments of neuronal development and synaptic communication observed (Dalla Massara et al., 2016). A decrease in the interaction between CBP and CREB has also been reported in the brains of postnatal mice with isoflurane-induced cognitive impairments, resulting from an increase in nuclear translocation of HDAC4. HDAC4 interacts with CREB in the nucleus, which results in an impairment in transcriptional activation of CREB and a decrease in the expression levels of BDNF and c-Fos (Sen and Sen, 2016). In an isoflurane-exposed maternal-fetal rat model, overexpression of HDAC2 induced the subsequent downregulation of CREB and was associated with spatial learning and memory impairments in the offspring (Luo et al., 2016). These changes were reversed by suberoylanilide hydroxamic acid (SAHA), a HDAC inhibitor marketed as Vorinostat which is FDA-approved for the treatment of leukemia (Witt et al., 2012), which was administered to the offspring before assessing learning and memory tested by the Morris water maze (Luo et al., 2016).

### Non-coding RNAs

Non-coding RNAs (ncRNAs) (Hombach and Kretz, 2016) represent a large and heterogeneous family of RNA molecules that do not encode proteins. Non-coding RNAs are loosely classified into two major classes: short (<200 nucleotides) and long (>200 nucleotides) ncRNAs. The description of multiple kinds of ncRNAs is exponentially increasing and it is now widely accepted that ncRNAs play major biological roles in a myriad of processes, ranging from embryonic development to aging. Micro RNAs (miRNAs) and long non-coding RNAs (lncRNAs) represent the best-characterized of the ncRNAs. Since they function as crucial regulators in gene expression, it is not surprising that dysregulations in miRNAs and/or lncRNAs activity are associated with many complicated human disorders including functional cognitive disorders caused by anesthetics.

Alterations in miRNA and lncRNA activity have been reported after inhaled and/or intravenous anesthetic exposure (Sun and Pei, 2015; Chen et al., 2016; Ye et al., 2016). These alterations may change the expression of related pathological intermediates in anesthetic-induced developmental neurotoxicity, suggesting that ncRNA-based signaling may be a novel target for preventing this neurotoxicity. One example is the neuronal microRNA, miR-124, which is upregulated in ketamine-induced neurodegeneration in mouse hippocampus (Xu et al., 2015). Knocking down miR-124 *in vitro* reduces ketamine-induced apoptosis in hippocampal CA1 neurons through upregulating α-amino-3-hydroxy-5-methyl-4-isoxazolepropionic acid (AMPA) receptor phosphorylation and activating PKC/ERK pathway (Xu et al., 2015). Mice subjected to hippocampal miR-124 inhibition *in vivo* showed improved memory performance (Xu et al., 2015). Another example is miR-21 which is down-regulated in propofol-treated human embryonic stem cell-derived neurons and regulates Sprouty 2 expression (Twaroski et al., 2014). A signal transducer and activator of transcription 3/miR-21/Sprouty 2/Akt-dependent mechanism is considered to be involved in propofol-induced cell death (Twaroski et al., 2014). BDNF antisense RNA (BDNF-AS) is one discovered functional lncRNA which inhibits the expression of BDNF (Modarresi et al., 2012). BDNF-AS is upregulated in ketamine-injured mouse embryonic neural stem cell-derived neurons, while BDNF is downregulated (Zheng et al., 2016). Downregulation of BDNF-AS protects neurons against apoptosis and promotes neurite outgrowth, possibly via the activation of the BDNF-TrkB signaling pathway (Zheng et al., 2016). Additional changes to ncRNAs are also reported in various models of anesthesia-induced neurotoxicity (Jiang et al., 2014; Cao et al., 2015; Sun and Pei, 2016; Song et al., 2017; Zhou et al., 2017).

Additionally, circular RNAs (circRNAs) belong to a new class of ncRNA molecules that are highly abundant in the brain and influence the regulation of gene expression (Hansen et al., 2013). Many circRNAs change their abundance abruptly corresponding to the timing of synaptogenesis (You et al., 2015). Studies have suggested that circRNAs may regulate synaptic plasticity and neuronal function (Szabo et al., 2015; van Rossum et al., 2016). Some studies have provided an insight into the function of circRNAs in neurodegenerative diseases, such as Alzheimer’s disease and Parkinson’s disease (Floris et al., 2016), and the neurotoxic effects observed in animals exposed to anesthetics. Evidence includes histological changes in neurodegenerative changes. These provide new insights into the possible association of circRNA dysregulation with anesthesia-induced neurotoxicity, although to date the literature is limited. For example, circRNAs can function as miRNA sponges (Hansen et al., 2013) and miRNAs involved in anesthesia-induced neurotoxicity may get inhibited by some unknown circRNAs.

### Epigenetic Crosstalk

In addition to the independent regulation by individual epigenetic mechanisms, it is interesting that an epigenetic crosstalk, i.e., interplay between DNA methylation and histone methylation (Du et al., 2015), may be involved in the processes of modulating disease-associated genomic loci and gene products. Collaborative activities of different epigenetic modifications could result in a common outcome, gene transcription or gene silencing. For example, methyl-CpG-binding protein 2 (MeCP2) is believed to function as a transcriptional repressor by binding to methyl-CpG, recruiting chromatin remodeling proteins, and further suppressing the expression of genes. MeCP2 integrates DNA methylation and histone acetylation at the BDNF gene suppression induced by anesthesia in neonatal rats via enhanced interaction with DNMT1 and HDAC2 (Wu et al., 2016).

## Conclusion

**FIGURE 1:**
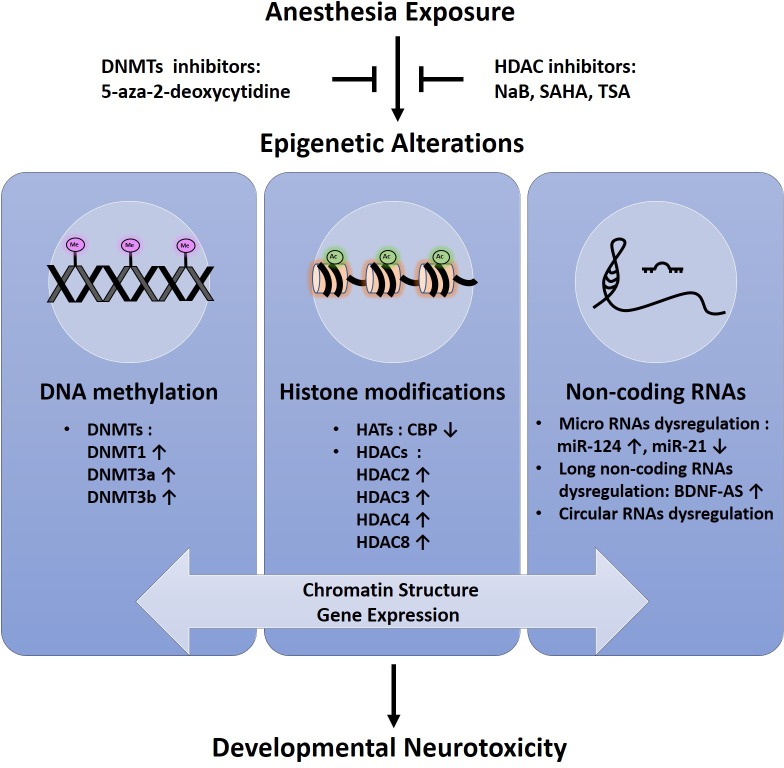
Schematic representation of epigenetic alterations in anesthesia-induced neurotoxicity in the developing brain.

So far, conflicting data exist about the effect of anesthetic agents on neurodevelopment in humans and no definite conclusion has been given yet. Although general anesthetics have been considered neuroprotective in pre-clinical studies (Nunes et al., 2013; Zaugg et al., 2014), the effect of anesthesia-related neurotoxicity remains an area of concern. The most recent studies suggest a novel epigenetic-related mechanism by which anesthetic-induced neuronal toxicity in developing human neurons and animal models (**Figure 1**). Success of therapeutic intervention using epigenetic modifiers such as DNMT inhibitors and HDAC inhibitors implicates that the epigenetic intervention is promising as potential targeted therapies aimed at mitigating neurotoxic effects of anesthetics in developing brain (**Figure 1**). Further research is needed to fully elucidate the epigenetic basis and its role in this field.

## Author Contributions

PZ and ZW wrote this review article.

## Conflict of Interest Statement

The authors declare that the research was conducted in the absence of any commercial or financial relationships that could be construed as a potential conflict of interest.
